# COVID-19 and Older Adults: Coping With Long-Term Pandemic Precautions

**DOI:** 10.1093/geroni/igab046.137

**Published:** 2021-12-17

**Authors:** Kerstin Emerson, Deborah Kim, George Mois, Jenay Beer

**Affiliations:** University of Georgia, Athens, Georgia, United States

## Abstract

Studies conducted at the beginning of Covid-19 precautions suggested that older adults were stressed, but hopeful. Less is known how coping has changed for older adults after experiencing months-long pandemic precautions. We explore differences in coping between the initial pandemic declaration in March 2020, and 9 months later, via an internet survey fielded in November 2020 (n= 781). We present summary data, using chi-square tests for subgroup analyses. A majority of respondents (aged M=66 yrs, range 60-89) were women (64%) and White (94%). When asked to compare their feelings to the beginning of the pandemic, 44.8% were more frustrated, 38.7% were more stressed, and 32.7% were more anxious. However, 38.3% were more appreciative. Women were significantly more likely than men to report increases in feeling frustrated, angry, scared, stressed, sad, and hopeless. Introverts were significantly more likely than extroverts to report an increase in loneliness and stress. Since the first few weeks of the pandemic, respondents reported more communication through video calls (45.2%), texting (40.2%), and phone calls (28.8%). Additionally, 61.5% spent more time on computers/tablets, 47.2% spent more time watching TV, and 24.5% spent more time praying. Extroverts were significantly more likely than introverts to report an increase in time with TV, phones, and computers/tablets. Women were significantly more likely than men to report increased texting and praying. These data provide further understanding of the impact of long-term pandemic precautions on older adults and suggest particular subgroups of older adults may benefit from public health and mental health interventions.

